# Study on Localized Surface Plasmon Coupling with Many Radiators

**DOI:** 10.3390/nano11113105

**Published:** 2021-11-18

**Authors:** Zhizhong Chen, Chuhan Deng, Xin Xi, Yifan Chen, Yulong Feng, Shuang Jiang, Weihua Chen, Xiangning Kang, Qi Wang, Guoyi Zhang, Bo Shen

**Affiliations:** 1 State Key Laboratory for Artificial Microstructure and Mesoscopic Physics, School of Physics, Peking University, Beijing 100871, China; deng121@stu.pku.edu.cn (C.D.); xixin@pku.edu.cn (X.X.); 1501110129@pku.edu.cn (Y.C.); fengyulong@pku.edu.cn (Y.F.); jiangshuang@pku.edu.cn (S.J.); chenwh@pku.edu.cn (W.C.); xnkang@pku.edu.cn (X.K.); wangq@pku-ioe.cn (Q.W.); gyzhang@pku.edu.cn (G.Z.); bshen@pku.edu.cn (B.S.); 2 Dongguan Institute of Optoelectronics, Peking University, Dongguan 523808, China

**Keywords:** localized surface plasmon, light emitting diode, dipole, many radiators, cathodoluminescence, perturbation method

## Abstract

Localized surface plasmon (LSP) coupling with many radiators are investigated. The LSP is generated by excitation of laser or electron beam on the random Ag nano particles (NPs) and arrayed ones embedded in the p-GaN of green LEDs. They couple with the excitons or radiative recombination in the quantum well (QW) and electron beam, which enhance or suppress the luminescence of the radiators. The photoluminescence (PL) intensity of periodic Ag NPs can get as much as 4.5 times higher than that of bare LED. In addition to the periodic structure, the morphology of Ag NPs also affects the localized SP (LSP) resonance intensity and light scattering efficiency. In the finite difference time domain (FDTD) simulation, five x-polarized dipoles are approximated to five quantum wells. Considering the interaction between the five dipoles and their feedback effect on LSP, the enhancement effect of SP dipole coupling with Ag NPs is amplified and the energy dissipation is reduced. The enhancement of cathodoluminescence (CL) was also found in green LEDs with Ag NPs. The three-body model composed of two orthogonal dipoles and an Ag NP is used for 3D FDTD simulation. The LSP-QWs coupling effect is separated from the electron beam (e-beam)-LSP-QW system by linear approximation. Under the excitation of electron beam, the introduction of z-dipole greatly reduces the energy dissipation. In the cross-sectional sample, z-polarized dipoles in QWs show more coupling strength to the dipole and quadrupole modes of LSP. The perturbation theory is used to separate the LSP coupling effects to x-dipole and z-dipole. At last, the resonator and the antenna effects are discussed for LSP coupling at different positions to the Ag NP.

## 1. Introduction

Surface plasmon (SP) can be coupled with excitons in radiators or photons in free space, and can be applied to high-efficiency, high-speed light-emitting diodes (LEDs) and color conversion [1,2,3,4]. In 1999, Gontijo et al. predicted that when SP was resonantly excited in metal nanostructures, the spontaneous emission rate (SER) of the radiator could be increased by more than 1000 times, and then its evanescent field had an impact on the radiator [5]. By exciting the SP gap mode of nano patch antenna (NPA), the SER of Ru dye is increased by 1000 times, and the quantum efficiency remains above 50% [6]. However, there is little room to improve the power efficiency of blue or white LEDs because their high external quantum efficiency (EQE) can approach to 80% [7]. It is well known that metal dissipation loss is large due to Ohmic loss, generation of electron hole pairs and high-order SP modes [5,8,9]. Therefore, when it is necessary to improve the EQE of LED, the luminous intensity enhanced by SP should be greater than that dissipated by metal. SP technology is an effective way to improve EQE of ultra-low efficiency radiator, such as long wavelength LED, deep ultraviolet (DUV) LED and LED with reduced efficiency at high injection level [10,11,12,13]. SP can also improve the bandwidth of LED in visible light communication (VLC) applications. Although it can be predicted theoretically that the modulation bandwidth of LED can achieve tens of GHz [2,14], in practical application, the bandwidth of SP has difficulty reaching twice the traditional modulation bandwidth of the conventional one [15,16]. It is hard to obtain the SP-enhanced devices with high SER and EQE practically.

The energy matching between the excitation or emission energy of the radiator and the resonance energy of SP should be satisfied. The material, size and morphology of metallic nanoparticles (NPs) will sensitively change the localized SP (LSP) resonance energy [17,18,19,20]. However, how they affect the energy transferring to scattering and metal dissipation is not clear. There are seldom reports on quantitative IQE and LEE of the LSP coupling system. Most metallic NPs are prepared by thermal annealing of metal films. Their sizes and morphology distribute in large range, which reduces the coupling strength of SP [20]. The periodicity of metal NPs is also important for the coupling of SP and radiator [21,22,23,24]. The fabrication of the periodically metallic NPs is difficult and expensive. When LSP is strongly coupled with radiator, metal loss should be considered. In general, the practical single metallic NP is used as a good resonator or antenna [9,25,26]. They exhibit high Purcell factor with high Ohmic loss [9], or enhance excitation and light extraction, but have no significant effect on the SER of the radiator [25,26]. It is important to build a metallic nanostructures with both resonator and antenna.

In addition to metallic nanostructures, the light source conditions also have a great influence on SP coupling. Gontijo et al. believed that the electron wave function in semiconductors was spatially extended, which helped to avoid metal dissipation [5]. InGaN/GaN multi quantum wells (MQWs) is a common active layer structure in LED epitaxial structure. GaN barrier is an intermediate layer between InGaN QWs with a thickness of about 10 nm. There will be 2 or 3 wells of MQWs in the evanescent field. When two or more QWs are coupled with the same LSP mode of metal NP, cooperative emission will be considered [27,28,29,30,31,32]. C. C. Yang et al. established theoretical formulas and numerical algorithms to evaluate the radiation power enhancement during the coupling of two radiation dipoles with LSP induced on nearby Ag NP [33,34,35,36]. They proposed the feedback effect of LSP evanescent field on radiation dipole behavior [37,38,39,40]. When the dipole is laterally away from the Ag NPs, the spectral peak of the enhanced radiation power redshifts, and its coupling level decreases with the increase of the transverse distance [37]. Two models of radial or orbital dipole coupling with LSP in Ag NPs reveal different mechanisms in simulation, which is mainly due to the difference of surface charge distribution of Ag NPs [38,40]. The radial dipole will couple to dipole mode of LSP, while the orbital dipole will couple to high-order LSP mode. In C-plane InGaN/GaN QWs, the radiating dipoles are mainly oriented in the plane of the QW [38]. So under the Ag NPs, the orbital dipoles is dominant and less coupling to LSP. In a semipolar QW structure, an out-of-plane dipole may exist [41,42,43]. However, few experimental results are reported on the LSP coupling to semipolar QW.

Recently, there have been several reports on the excitation of SP in metallic NPs by electron beam (e-beam) through cathodoluminescence (CL) [44,45,46,47]. Because of its ultra-high spatial resolution and wide-band optical sensitivity, it can be used to study the optical process in nanostructures. Some researchers regard the e-beam as a dipole along the incident direction in CL measurement [44,47]. The vertical dipole will produce high-intensity near-field for the Ag NPs, which will significantly affect the coupling between LSP and QWs. The dipoles representing quantum wells and e-beam are in-plane and out-plane, respectively. The configuration of single Ag NP and two orthogonal dipoles can be used to study the energy transfer process of LSP-QW coupling system. If the Ag NPs are on the cross-section of the LED wafer, the polarization of dipole will be in an arbitrary direction. In this case, LSP will coupling to the arbitrary polarization dipoles. The perpendicular dipoles corresponding to electron beam provide a new chance to study the LSP coupling to many radiators.

When the radiators interact with each other through the LSP, the coupling effect on each dipole can hardly be separated from the whole system. Therefore, the energy transferring in the individual dipole and its corresponding LSP radiation and dissipation energies are not known [23]. A perturbation method is proposed, which is a set of methods for studying various problems in mathematics, mechanics and physics [48,49,50]. In this work, many radiators coupled with an LSP mechanism on different LED structures were studied by photoluminescence (PL), CL measurements and three-dimensional (3D) finite difference time domain (FDTD) simulation [51]. The radiators generally refer to QWs or QDs, which can be approximated by dipoles. Although the electron beam does not emit light, it has energy to excite LSP, so it can also be approximated by dipoles [44,47]. In FDTD, the polarization orientation of dipoles can be defined, and the luminous range of dipoles can also be defined. The luminous range is a little wider than the actual peak width at half height. In Section 2, the traditional InGaN/GaN multiple QWs are simplified to five parallel dipoles. The size and morphology of Ag NPs were optimized by the scattering and dissipation decay rate of LSP [22]. According to the initial quantum efficiency (IQE) and FDTD simulation results of bare LED, the IQE and light extraction efficiency (LEE) of LSP-coupled LED are deduced respectively. Considering the interaction between the five dipoles and their feedback effect on LSP, the LSP-dipoles coupling is enhanced and the energy dissipation is reduced [23]. In Section 3, the coupling between electron beam (e-beam) induced LSP and QWs is studied [12,52,53]. CL measurements were carried out on Ag-MQWs system. The coupling mechanisms between orthogonal dipole and LSP, arbitrary polarization dipole and LSP in green LED are simulated by FDTD. The three-body system of e-beam-LSP-QW is demonstrated by linear approximation and perturbation method. Finally, the coupling between LSP and many radiators is summarized, and the new metallic NPs/LED structure is predicted.

## 2. Localized Surface Plasmon Coupling to Parallel Quantum Wells

### 2.1. Ag NPs Hexagonal Arrays Embedded in LEDs and Their Photoluminescence (PL)

Green LED structures were grown on c-plane sapphire substrates by metal organic chemical vapor deposition (MOCVD). There were five InGaN/GaN QWs (2.5 nm/12.5 nm) in the green LED, which were covered with a 160 nm thick p-GaN layer. SiO_2_ thin films with a thickness of 150 nm were deposited on bare green LED by plasma enhanced chemical vapor deposition (PECVD). An Eitre^®^ 3 Nanoimprint instrument (Eitre^®^ 3, Lund, Sweden) was used to conduct a two-step simultaneous thermal and ultraviolet curing (STU) imprint process. Then periodic Ag NPs were prepared by nano imprint lithography (NIL) and induced coupled plasma (ICP) etching, which were embedded into the hexagonal nanohole array of p-GaN. The depth of the nanoholes is about 150 nm, indicating that the Ag NPs are 10 nm away from the top of the QW. NIL technology is suitable for large-scale production of nano patterns [54]. The size and morphology of Ag NPs depend on the etching time of SiO_2_, the thickness of Ag film deposited on the pattern which subsequent goes through thermal annealing process. Details of SiO_2_ etching and silver film thermal annealing are given in Ref. [22]. Scanning electron microscopy (SEM, Nova Nano SEM 430 produced by FEI company, Hillsborough, OR, USA) were used to study the surface morphology of Ag NP array LEDs. The room temperature PL measurements were performed on the Ag NPs embedded LEDs and the reference samples by a 405 nm laser. Both the excitation and detection were carried out on the top surface of the LEDs.

**Figure 1:**
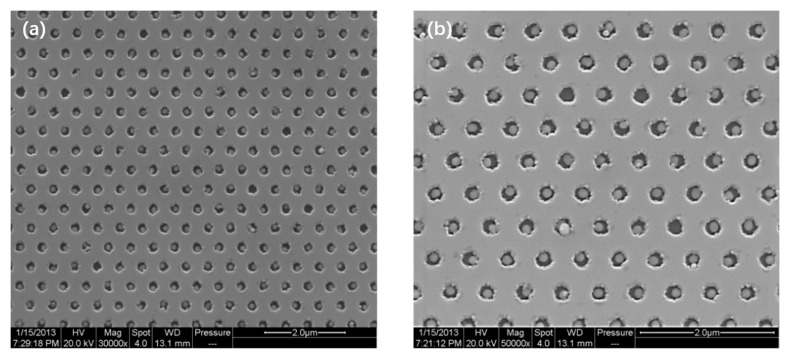
Top view SEM images of Ag NP array LEDs with the size of (**a**) 90 nm and (**b**) 180 nm on green LED epitaxial wafer. For hexagonal array nanoholes in p-GaN cladding, each hole is filled with a single Ag NP.

Figure 1 shows the top view SEM images of Ag NP array embedded LEDs. The period of the Ag NP hexagonal array is 545 nm. In Figure 1a,b, the sizes of Ag NPs are 90 and 180 nm, respectively, with nanohole diameters of 190 and 250 nm. In the nanohole, the Ag NP is usually a spherical cap. The NP shape is characterized by the aspect parameter α = h/r, where h represents the height of the spherical cap and r represents its radius. In the cross-sectional SEM images (not shown here), the α values were measured as 1.78 and 1.00 for 90 nm and 180 nm Ag NPs, respectively [22]. The samples with 90 and 180 nm Ag NPs are 90nm Ag-PhC-LED and 180 nm Ag-PhC-LED, respectively. As the reference samples without Ag NPs, the samples with same period of 190 and 250 nm photonic crystals (PhCs) are named by 190 nm PhC-LED and 250 nm PhC-LED. The virgin LED without any structures is named as bare LED.

**Figure 2:**
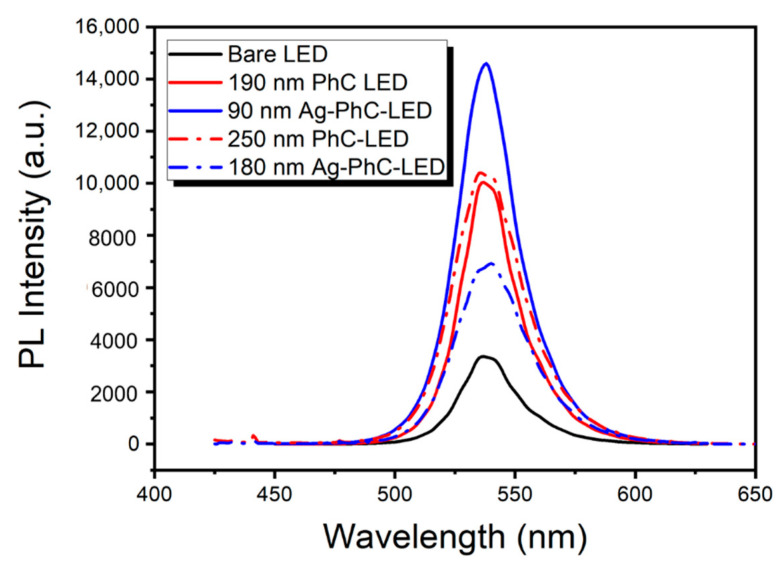
PL spectra for the samples of Ag NPs embedded LED (90 nm Ag-PhC-LED and 180 nm Ag-PhC-LED), photonic crystal LED (190 nm PhC-LED and 250 nm PhC-LED) and bare LED.

The PL spectra are measured on the samples of Ag NPs embedded LEDs, photonic crystal LED and bare LED, as shown in Figure 2. PL intensity for 90 nm Ag-PhC-LED enhances 4.5 times compared to the bare LED, while for 180 nm Ag-PhC-LED it only enhances 2 times. As to the PhC-LED samples, the PL enhancements are nearly same at 3 times compared to the bare LED one. It indicates that 90 nm Ag NPs play more roles in the light extraction and/or LSP resonantly coupling than 180 nm Ag NPs. In Ref. [22], the time resolved PL (TRPL) results of these samples show that LSP-MQWs coupling strength of 90 nm Ag-PhC-LED enhances about 36% compared to that of 180 nm one. The 180 nm Ag NPs may cause more energy dissipation, which leads to less PL intensity than that for PhC LEDs. The PL enhancement or suppression will be simulated by 3D-FDTD in the following.

### 2.2. FDTD Simulation for LSP Coupling to MQWs in LED

**Figure 3:**
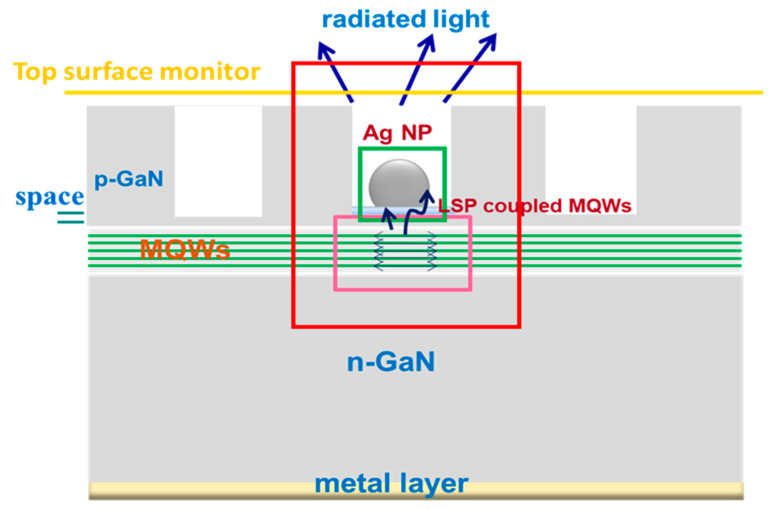
Schematic structure of Ag NP arrayed LED in 3D FDTD simulation. The five dipoles located below Ag NP represent five InGaN QWs. The pink frame is used to collect the total power radiated by the five dipoles. The green frame is used to detect the dissipated power in Ag NP. The red box is used to collect scattered energy from all directions of the system. The gold line is used to record the upward radiated power. Except for the substrate reflection layer with metal boundary conditions, all sides adopt a perfectly matched layer (PML) absorption boundary.

According to the Ag NPs in the green LED in the above SEM images, the FDTD model is established, and the PL experiments are simulated to study the LSP enhanced PL mechanism. The interaction between adjacent Ag NPs can be ignored because their spacing is greater than 100 nm, and the coupled Ag NPs should have a small gap less than tens of nanometers [9]. There is an Ag spherical cap in the central hole of hexagonal PhCs on p-GaN. Except for the substrate reflection layer with metal boundary conditions, all other surfaces adopt a perfectly matched layer (PML) absorption boundary. Considering that there is only a 10 nm p-GaN spacer between the Ag NPs and the topmost QWs, all five QWs are represented by five parallel dipoles at 10, 25, 40, 55 and 70 nm, respectively. The adjacent dipoles are very close to each other. There are three dipoles at the penetration depth of 40 nm [31]. When the other two dipoles are placed above 40 nm, they are less affected by SP. The FDTD simulation domain is 6 μm × 6 μm × 1.5 μm (x, y, z direction), which has quite fine spatial resolution under the maximum computing power. As shown in Figure 3, four monitors are used to detect electromagnetic (EM) power distribution. The pink frame monitor is used to collect the total power radiated by five dipoles (*P*_5−*dp*_). The green box monitor is used to detect the dissipated power *P_diss_* in Ag NP. The red frame monitor is used to collect scattered energy from all directions of the structure *P_scat_*. The last gold line monitor is on the upper surface to record the upward radiated power *P_up_*.

**Figure 4:**
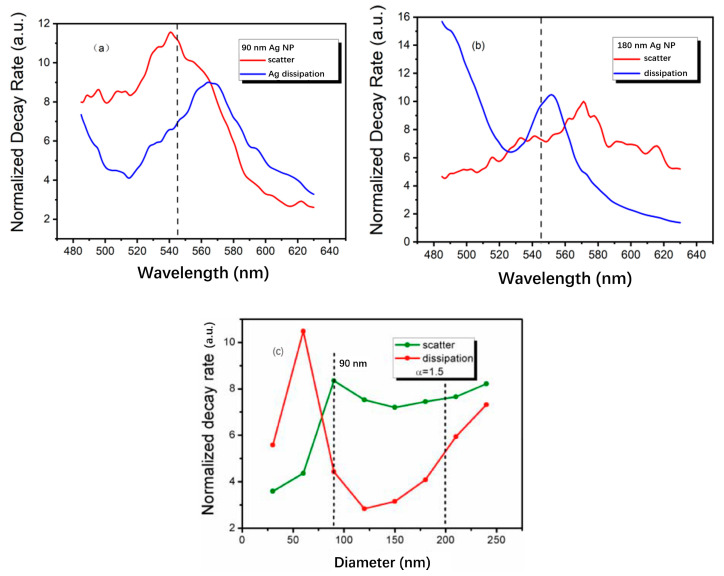
The normalized decay rate spectrum for (**a**) 90 nm Ag-PhC-LED, (**b**) 180 nm Ag-PhC-LED. The dash lines correspond to the 545 nm. (**c**) The normalized decay rate at 545 nm against Ag NP′s diameter, where the α is 1.5 [22]. (The normalized decay rates are normalized by the total energy of all the dipoles.). Reprinted with permission from ref. [22] 2015, IEEE.

In FDTD simulation, radiation is only way to consume the dipole power [51]. Some of them dissipate in the Ag NP. Others scatter out from the system of Ag-MQWs. The two channels consume the radiated dipole power. The decay rate is equivalent to the radiated or dissipated power in an inhomogeneous environment. Figure 4 shows the decay rates of the dissipation and scattering energies for different size of Ag NP. In Figure 4a, the decay rate of scatter and dissipation are about 11.2 and 6.8, respectively, at the wavelength of 545 nm for 90 nm Ag-PhC-LED. That the scatter decay rate is larger than the dissipation one causes more light emission enhancement. In Figure 4b for 180 nm Ag-PhC-LED, the decay rate of scatter and dissipation are about 7.3 and 9.7, respectively at the wavelength of 545 nm. It will lead to the PL suppression. The proportion of the dissipated energy to radiated dipole energy determines the PL emission enhancement or suppression. The diameter of Ag NPs in LEDs with a wavelength of 545 nm is optimized as shown in Figure 4c. The α is set to 1.5 in Figure 4c, which is a typical value in the annealing process of Ag film. It is worth noting that a smaller dissipation decay rate indicates more PL enhancement [20]. In Figure 4c the Ag NPs with a diameter in the wide range of 90 nm to 200 nm and α greater than 1.5, show higher scattering decay rate compared to the dissipation one, which may lead to significant light emission enhancement.

After SP is coupled with five QWs, the internal quantum efficiency (IQE) and light extraction efficiency (LEE) are improved at the same time. The IQE and LEE can be calculated for green LED by above FDTD simulation results. Considering that the initial IQE of green LED is about 26%, the non-radiative composite power of *P_non_* is about three times that of the radiative composite power *P_rad_*. In the process of SP coupling, the radiation power of the five dipoles increases from *P_rad_* to *P*_5−*dp*_, which is the sum of *P_scat_* and *P_diss_*. It is supposed that LSP-MQW coupling less influence the nonradiative recombination rate of the quantum wells [55]. IQE for the LSP enhanced LED is defined as the ratio of radiative recombination to the global recombination, which can be calculated as,

(1) $$\mathrm{IQE}=\frac{P_{scat}}{P_{non}+P_{5-dp}}$$

Combined the time resolved PL (TRPL) and IQE measurements, the *P_diss_* can be measured according to Equation (1). The PL signal is detected on the top surface of the LED wafer. The LEE is defined as the ratio of the light extraction from top surface to the whole scattered light in the semiconductor. The LEE is determined by the total upward radiation power *P_up_* to *P_scat_* of the coupling system. The equation is described as,

(2) $$\mathrm{LEE}=\frac{P_{up}}{P_{scat}}$$

According to Equations (1) and (2), the external quantum efficiency (EQE) of the green LED can be calculated as,

(3) $$\mathrm{EQE}=\mathrm{IQE}\;\times \;\mathrm{LEE}=\frac{P_{up}}{P_{non}+P_{5-dp}}$$

**Figure 5:**
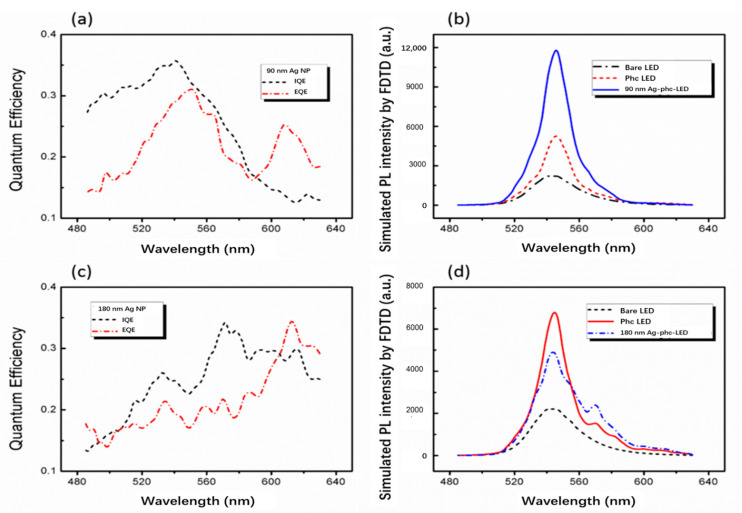
The FDTD simulated (**a**) quantum efficiency spectra, (**b**) PL spectra for 90 nmAg-PhC-LED with α = 1.78 and the correlative samples. Reprinted with permission from ref. [23] 2016, Springer Nature. The FDTD simulated (**c**) quantum efficiency spectra, (**d**) PL spectra for 180 nm Ag-PhC-LED with α = 1 and the correlative samples.

Figure 5 shows the FDTD simulated IQE, LEE spectra and PL spectra of 90 nm- and 180 nm-Ag-PhC-LED samples. Figure 5a,c show the IQE and LEE spectra of two Ag-PhC-LED samples. For 90 nm-Ag-PhC-LED sample, the IQE at 545 nm is 34.7%, which is significantly higher than 26% of the original sample. However, the IQE of 180 nm-Ag-PhC-LED sample at 545 nm is only 23.5%. The LEE of 90 nm-Ag-PhC-LED samples is 30.1%, which is also greater than 17.9% of PhC-LED samples without Ag NPs. For 180 nm-Ag-PhC-LED samples, the LEE is 18.6%, which is less than 23.5% of PhC-LED samples. Although the spontaneous emission of the five dipoles has been enhanced, the IQEs have not been enhanced certainly due to the dissipation of metal. The LEEs of the samples are determined by the influence of LSP and PhCs on the waveguide modes. Obviously, the low α of Ag NPs cannot extract the waveguide mode out from the LEDs. Figure 5a,c also show that the values of IQE and LEE depend on the wavelength. The 90 nm-Ag-PhC-LED sample enhanced both IQE and LEE at 545 nm, while the 180 nm-Ag-PhC-LED suppressed both IQE and LEE. For long wavelengths, for example, at 620 nm, IQE and LEE are greater than those of PhC-LED samples and 90 nm-Ag-PhC-LED samples. Therefore, it is necessary to carefully design the morphology of PhC hole array and Ag NPs at a certain emission wavelength.

Assuming the IQE of PhC-LED and bare LED do not change, the PL spectra in Figure 5b,d are calculated according to the above IQE and LEE spectra results, which are similar to the experimental results shown in Ref. [22]. The PL spectra of bare LEDs are the experimental ones, which are used to calibrate the calculated PL spectra of Ag-PhC-LEDs and PhC-LEDs by their IQE and LEE spectra. The PL intensity of 90 nm-Ag-PhC-LED sample is about twice that of PhC-LED sample. For 180 nm-Ag-PhC-LED samples, the PL intensity is 1.5 times lower than that of PhC-LED sample without Ag NPs. These results are in good agreement with the experimental results. Compared with the experimental results, there is a certain error in PL enhancement factor. Simulation approximation includes dipole approximation of MQWs [40,41,55], dipole position and polarization approximation [37,38] and single Ag NP approximation and simplification of simulated LED model approximation [9,51]. In conclusion, IQE, LEE and PL simulations provide some energy transfer information for the coupling of LSP and MQWs. However, in the above simulation the five dipoles couple to LSP as a whole. It is not well known how the interaction between dipoles influences the LSP coupling system.

**Figure 6:**
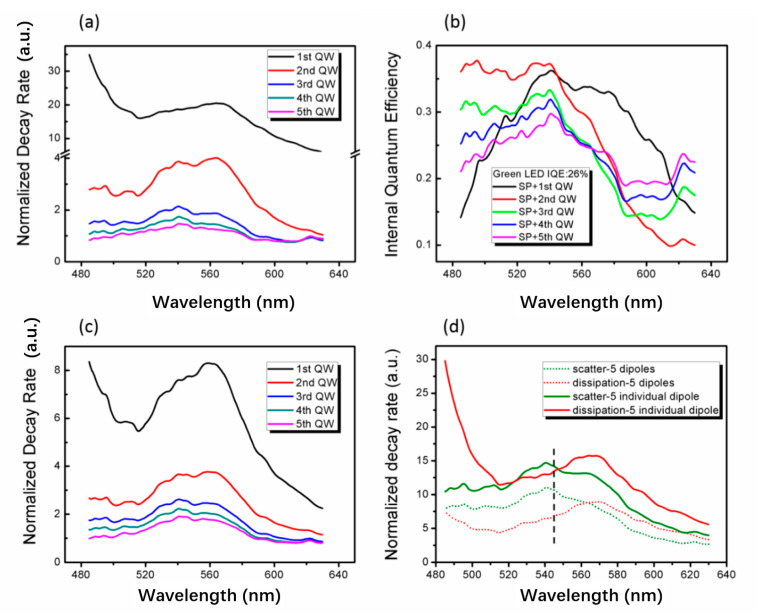
In FDTD simulation of 90 nm-Ag-PhC-LED sample, (**a**) normalized decay rate curve of single dipole on different quantum well layers. Reprinted with permission from ref. [23]. 2016, Springer Nature (**b**) IQE curves of SP with single dipole coupling system, (**c**) normalized decay rate curve of five dipoles on different quantum well layers at the same time, (**d**) Normalized decay rate curves of scattering and dissipation of five combined dipoles and five independent dipoles. Reprinted with permission from ref. [23]. 2016, Springer Nature.

Figure 6 shows the FDTD simulation of LSP coupling of single dipole and combined dipoles, respectively. Here, the decay rate corresponds to the scattered or dissipated power, as described in Ref. [23]. In Figure 6a, the dipole decay rate at the first QW is as fast as 18.8 at the wavelength of 545 nm and about 1.0 without LSP coupling. The second QW is 3.8. It is consistent with the decay law of LSP dipole coupling strength with distance. For the fifth QW, the decay rate fluctuates around 1.0, that is, the influence of LSP dipole coupling can be ignored beyond the fifth QW. Figure 6b shows the IQE curves of LSP coupled with the single QW. From the first QW to the last QW, the IQE at 545 nm are 35.2, 36.0, 31.5, 30.2 and 29.1%, respectively. The IQE of the second QW is higher than that of the first QW. This may be because more energy is coupled to the higher-order LSP mode and dissipated at a smaller distance, so the IQE decreases [55]. Figure 6c shows the normalized dipole decay rate of five dipoles placed in five QWs at the same time. It is found that the decay rate of the first QW at 545 nm is 7.7, which is much smaller than that of a single QW in Figure 6a. The decay rate curves of the other four QWs are similar to Figure 6a. Therefore, due to the existence of Ag NP, the interaction between five dipoles and Ag NP can occur within the penetration depth of LSP. This interaction leads to a decrease in the decay rate of the first QW. Figure 6d shows the scattering and dissipation decay rate curves of five independent dipoles and five combined dipoles, respectively. Although the scattering power of five-combined-dipole coupled LSP is less than that of five-independent-dipole coupled LSP, the proportion of scattering power to dissipated power at 545 nm is much greater than that of five-independent-dipole coupled LSP. In fact, the average IQE of the five independent dipoles is 32.4%, which is less than the combined dipoles of 34.7%. The LEE for five independent dipoles is 27.5%, which is also less than 30.1% of the combined dipoles. This means that the dynamic feedback effect of LSP coupling on combined dipoles can amplify their coupling strength.

**Figure 7:**
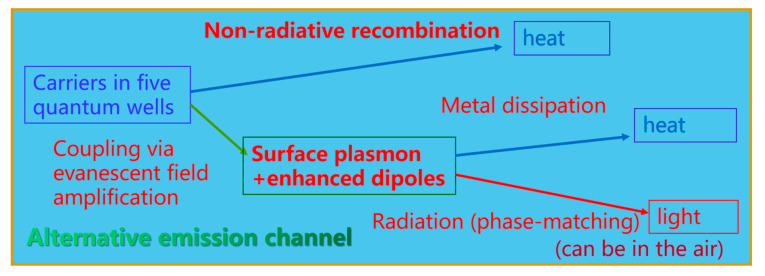
The surface plasmon and quantum wells coupling model. Reprinted with permission from ref. [23] 2016, Springer Nature.

According to the PL measurements and FDTD simulations on the Ag-PhC-LED samples, the energy transferring paths are considered in the LSP-MQWs coupling system. Figure 7 shows the surface plasmon and quantum wells coupling model. Here non-radiative recombination is assumed not to be influenced by LSP-MQW coupling [56]. Therefore, its energy directly transfers to heat. The radiative recombination excites LSP and LSP enhanced the radiative recombination conversely. The coupling system includes LSP and enhanced dipoles. The phase-matching modes are radiated as light emission, and others are metal dissipation as heat. Many researchers believe that the LSP coupling with radiators only enhance the light emission for the low efficiency devices [10,11,12,13]. It is easily understood that the non-radiative recombination is less for high efficiency devices. The efficiency is hard to enhance because the metal dissipation cannot be suppressed effectively. However, more energy enters into the evanescent field, the radiative recombination rate will be enhanced and amplified. More carriers in the quantum well will effectively conduct radiative recombination, which means the original non-radiative recombination will be reduced. Furthermore, the dissipation can be suppressed effectively for many parallel QWs coupling to LSP. Both IQE and LEE can be enhanced more by plasmonic cooperative emission [27,28,29,30]. It is reasonable that even if the EQE is as high as above 80% for common blue LEDs, above 95% EQE may be achieved if the structures of active layer and metallic NPs are well designed.

## 3. Surface Plasmon Coupling to Many Non-Parallel Dipoles

### 3.1. Perpendicular Dipoles

Recently, there have been several reports on the excitation of LSP in metallic nanoparticles (NPs) by electron beam (e-beam) through cathodoluminescence (CL) [45,46,57]. Because of its ultra-high spatial resolution and wide-band optical sensitivity, it can be used to study the optical process in nanostructures. Some researchers regard the e-beam as a dipole along the incident direction in CL measurement [45,57]. The vertical dipole will produce high-intensity near-field near the Ag NPs, which will significantly affect the coupling between LSP and QWs. The dipoles representing quantum wells and e-beam are in-plane and out-plane, respectively. The configuration of single Ag NP and two orthogonal dipoles can be used to study the energy transferring process of LSP-QW coupling system.

The samples are prepared similar to the Section 2.1. SEM and PL measurements are also carried out under the same condition. The CL test device used in this review is Quanta 200FEG environmental scanning electron microscope produced by American FEI company (Hillsborough, OR, USA) equipped with CL accessories. Its CL test accessories are Mono-CL4 system produced by Gatan company (Pleasanton, CA, USA), which can be used for CL test at normal temperature and low temperature. The resolution is 2 nm in high vacuum mode. CL measurement was carried out with an accelerating voltage of 15 kV and a beam current of 158 pA.

**Figure 8:**
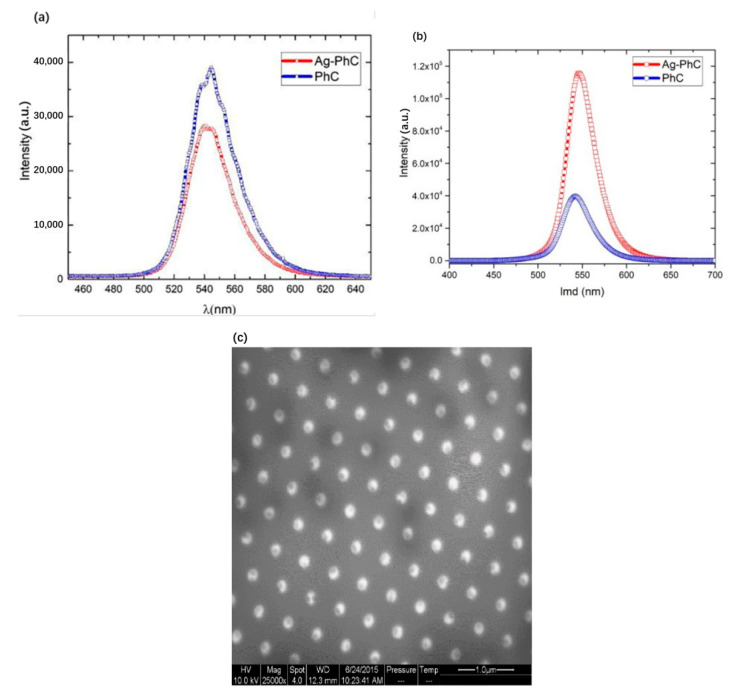
(**a**) PL spectra and (**b**) CL spectra of Ag-PhC-LED and PhC-LED samples. (**c**) Panchromatic CL image of Ag-PhC-LED sample.

In SEM images (not shown here), the diameter and height of the Ag NPs are about 160 nm and 80 nm, respectively. The diameter of p-GaN hole is about 250 nm. Figure 8 shows the PL and CL spectra of Ag-PhC-LED and PhC-LED samples. Compared with the PhC sample, the PL peak intensity of Ag–PhC-LED is reduced by 1.7 times, as shown in Figure 8a. This decrease is due to the large energy dissipation of Ag NPs as a result of their small aspect parameter α. The ratio of radiation energy to dissipation energy determines whether the final PL intensity is enhanced or suppressed. Surprisingly, the CL peak intensity of Ag-PhC-LED is 2.91 times greater than that of PhC-LED sample. The depth of electron penetration into the Ag NPs was more than 20 nm. Compared with laser excitation, e-beam excitation increases the relative emission intensity of Ag-PhC-LED samples by 4.95 times to that of PhC-LED ones. Considering the penetrating electron energy loss of Ag NPs, even if all electrons can penetrate Ag NPs and directly excite the quantum well, the enhancement factor cannot be as high as 4.95. In the process of LSP–QW coupling, the high near-field intensity of LSP in Ag NPs caused by continuous injection e-beam should be considered [53,54,55]. In Figure 8c, the black areas in the nano-holes are much smaller than the Ag NPs’ sectional areas. This also means that when LSP is excited in Ag NPs, the LSP can also excite QW to produce the CL emission.

**Figure 9:**
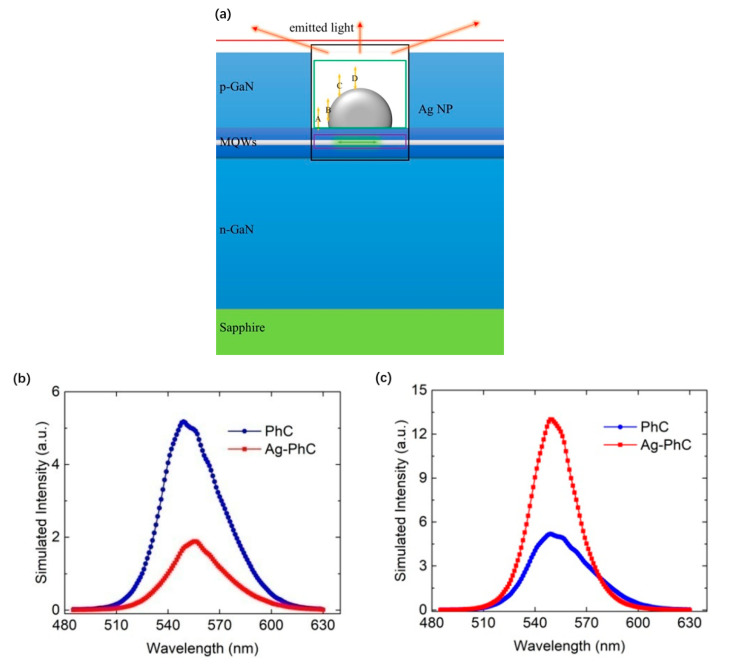
(**a**)The schematic structure of the Ag–PhC-LED sample in 3D finite difference time domain (FDTD) simulation. CL simulations were performed at e-beam impinging points A, B, C and D, respectively. The purple, green and black boxes are used to collect the total power radiated by the dipole, the dissipation power in the Ag NP, and the scatted energy, respectively. The red line (plane) is used to record the radiated power from top surface [12]. Simulated (**b**) PL spectra and (**c**) CL spectra for Ag-PhC-LED and PhC-LED samples. Reprinted with permission from ref. [12]. 2018, Multidisciplinary Digital Publishing Institute.

In order to distinguish different LSP-QW coupling mechanisms by e-beam excitation and laser excitation, 3D-FDTD numerical simulation is carried out. Figure 9a shows the schematic structure of Ag–PhC-LED sample used in 3D-FDTD simulation. The spacing between Ag NPs and the first QW is 10 nm. Perfectly matched layer (PML) absorption boundary is adopted. In order to improve the simulation accuracy, the mesh size of 2 nm is applied to the area of Ag NPs and x-dipole. Outside this area, an automatic hierarchical mesh is used. In addition, the simulation span is 6 μm × 6 μm (x–y plane), which is sufficient for light propagation. Considering the symmetry of the QW plane, where the radiating dipoles lie, only one dipole polarized along the x direction (x-dipole) was placed below the Ag NP to represent the QW. As mentioned in Section 2, the interaction between the QWs will influence the LSP-MQW coupling. However, due to the non-optimal distance between QWs, the influence is not very significant on the LSP-MQWs. In order to simplify the model, only one x-dipole is used to represent QW. The e-beam is represented by a dipole polarized along its trajectory(z-dipole) referring to [45,57]. Since the impact of e-beam on different positions of Ag NP may lead to different excitation, z-dipoles are placed at positions A, B, C and D with an interval of 30 nm, as shown by the yellow arrows in Figure 9a. As for PL simulation, since Ag NP is opaque to light excitation [58], or the laser wavelength cannot match the SP resonance wavelength [20], z-dipole is not included, which is similar to our previous work [22,23]. In order to calculate the power transition, four monitors were used in the simulation. The purple, green and black boxes are used to collect the total power radiated by the dipole, the dissipation power in the Ag NP, and the scatted energy, respectively. The red line (plane) is used to record the radiated power from top surface [12].

Figure 9b,c shows the simulated PL and CL spectra of Ag–PhC-LED and PhC-LED samples. The PL and CL experimental spectra of bare LEDs are used to calibrate the calculated PL and CL spectra of Ag-PhC-LEDs and PhC-LEDs by their IQE and LEE spectra. Figure 9b shows that the intensity of Ag–PhC–LED samples is reduced by 2.5 times, which is consistent with the experimental results. The Purcell factor at 545 nm was calculated to be 18.7, which shows that the LSP-QW coupling is very strong and the spontaneous emission rate is greatly improved. However, due to the dissipation of Ag NPs, the IQE of Ag-PhC sample is only 54% of that of PhC sample at 545 nm. In addition, the LEE of Ag-PhC is also reduced by 1.8 times. It is obvious that the decrease of IQE and LEE leads to the suppression of PL intensity.

CL simulations were performed at e-beam impinging points A, B, C and D, respectively. Considering that the electron beam itself does not radiate light, the energy radiated by the z-dipole must be subtracted after adding the z-dipole to simulate the impact of the electron beam at a specific impinging point. When the z-dipole and x-dipole exist at the same time, the relationship between the net power (named P_g_, P_p_, P_b_ and P_r_ respectively) flowing through the green box, purple box, black box and the upper red plane monitor in Figure 9a is,

*P_r_* = *P_up-x-dp_* + *P_up-z-dp_* (4)

*P_p_* = *P_x-dp_* (5)

*P_g_* = *P_z-dp_* − (*P_diss-x-dp_* + *P_diss-z-dp_*) (6)

*P_b_* = *P_x-dp_* + *P_z-dp_* − (*P_diss-x-dp_* + *P_diss-z-dp_*) (7)

where *P_x-dp_* and *P_z-dp_* are the power radiated by x-dipole and z-dipole, which can be directly recorded in the simulation. *P_up_* and *P_dis_*_s_ can also be recorded directly. They include x-dipole and z-dipole components, namely *P_up-x-dp_*, *P_up-z-dp_*, *P_diss-x-dp_* and *P_diss-z-dp_*. In order to distinguish the efficiency of x-dipole (QW) and two orthogonal dipole systems, each point is simulated without x-dipole. It is known that the electron energy is about tens of keV, the photon energy is about several eV in QW. The energy of the e-beam cannot be completely converted into QW radiation. The ratio of the amplitude of the z-dipole to the amplitude of the x-dipole is approximately set to be 10, considering power of the dipole is directly proportional to the square of its amplitude. The simulation results show that the existence of x-dipole has little effect on z-dipole, while z-dipole has a great effect on x-dipole by exciting LSP. We set,

*P_z-dp_* = (1 + *β*)*P′_z-dp_* (8)

where prime (′) represents all the power recorded without the x-dipole and *β* is a small quantity Therefore, it is reasonable to assume that the dissipated power and extracted power of the z-dipole vary linearly with *P_z-dp_*, that is,

*P_diss-z-dp_* = (1 + *β*)*P′_diss-z-dp_* (9)

*P_up-z-dp_* = (1 + *β*)*P′_up-z-dp_* (10)

*P_diss-x-dp_* = *P_g_* − (1 + *β*)*P_g_*′ (11)

*P_up-x-dp_* = *P_r_* − (1 + *β*)*P_r_*′ (12)

Based on Equations (1)–(3), (5), (11) and (12), the EQE of the x-dipole (QW) in e-beam-LSP-QW system is obtained. The typical impact positions A, B, C and D in the Ag-PhC samples were calculated, and their weights were considered by their Purcell factors. Figure 9c shows the averaged CL spectrum of Ag–PhC samples. Compared with PhC-LED samples, the CL strength of Ag-PhC-LED samples is increased by 2.4 times, which is consistent with the experimental result (2.91 times). It is found that except for the central region, the EQE of x-dipole in most regions around Ag NP is increased by more than 2 times. The EQE at position D is only 0.27 times that of the PhC sample, which is consistent with the panchromatic CL image in Figure 8c. In FDTD simulation, two orthogonal dipoles are coupled with LSP. The x-dipole effect in e-beam-LSP-QW system is separated by linear approximation method. The simulation results show that IQE and LEE are enhanced by adding z-dipole to LSP-QW system.

### 3.2. Dipoles at Arbitrary Polarization Orientation

**Figure 10:**
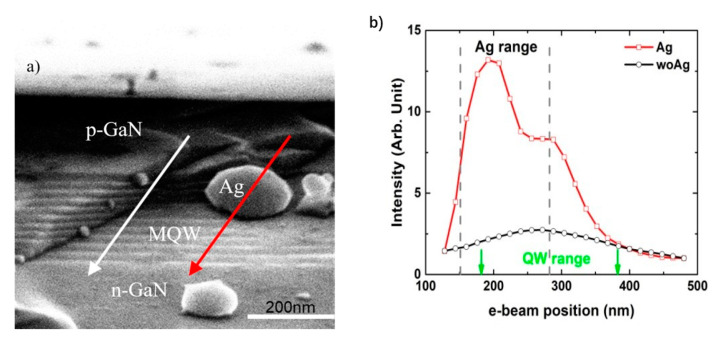
(**a**) Cross-sectional SEM image of LSP-QW coupling sample. Ag NP overlaps with QWs/p-GaN region. The long axis and short axis of Ag NP are 200 and 120 nm respectively. (**b**) The relationship between the peak intensity and position of CL line scanning. The red and black curves correspond to the red and white lines in (**a**). Reprinted with permission from ref. [53]. 2018, the Royal Society of Chemistry.

Although the perpendicular dipoles can enhance IQE and LEE for LSP-QWs system, the effect of the polarization orientation on the LSP-many radiator is still unknown. When the samples are cracked to show the cross section, the arbitrary polarization orientation to the e-beam incident direction may appear. The Ag NPs embedded in the hexagonal PhCs array holes were fabricated in a green LED by nanoimprint and lift-off techniques as Section 3.1. Cross section samples were prepared by cutting and cleavage for SEM and CL measurements. Some Ag NPs were dispersed in PhC holes and located in QWs region. CL line scan measurements were performed with and without Ag NPs. As shown in Figure 10a, Ag NPs were prepared on the cross section of green emission QWs. By setting the e-beam incident direction parallel to the quantum well plane, the effect of dipole polarization direction on LSP-QW coupling can be studied. As shown in Figure 10b, Move the e-beam (electron energy of 10 keV) along the white line and red line, and measure the CL spectrum of each point. The scanning step is set to be 16 nm. The red line scans through the Ag NP of p-GaN to QWs (labeled Ag), and the white parallel line scans through the area without Ag NP (labeled woAg). When the electron collision point is not in the range of Ag NP or QWs, the CL peak intensities of the two spectral lines are similar. When the e-beam approaches to Ag NP, the intensity increases rapidly. The maximum CL peak intensity is obtained near the top of Ag NP, which is 6.1 times higher than that without Ag NP at the same position. The enhancement of CL intensity is attributed to LSP caused by high-energy electron beam and/or excited QWs.

**Figure 11:**
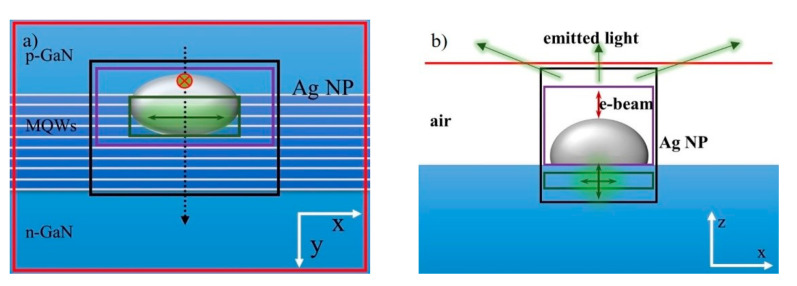
(**a**) The top view and (**b**) the side view of the schematic structure in the 3D FDTD simulation. The total radiation power of q-dipole, the dissipation power and scattering power of Ag NP are collected by green box, purple box and black box respectively. The red plane monitor is used to record the power emitted from the top surface. The black dashed line corresponds to the e-beam scanning path [53]. Reprinted with permission from ref. [53]. 2018, the Royal Society of Chemistry.

As shown in Figure 11, each QW is represented by a series of point dipoles (q-dipoles) polarized in the QW plane (x–z plane). Considering the symmetry of the q-dipole orientation in the QW plane, the polarization angle between the q-dipole orientation and the *z*-axis is set to 0° to 90°. Dipoles with orientations of 0° and 90° correspond to radial and orbital dipoles [38]. To simplify the model, the e-beam is represented by another point dipole (z-dipole) polarized along its trajectory (*z*-axis), as shown by the red dipole in Figure 11. By continuously moving the z-dipole along the black dotted line (corresponding to the red line in Figure 10a), the CL line scanning with Ag NP can be simulated.

The Purcell factor and EQE of each QW at different polarization angles are simulated. With the increase of polarization angle from 0° to 90°, the Fp of q-dipole at 270 nm decreases monotonically from 100 to 28.7, and the Fp of q-dipole at 210 nm decreases from 37.2 to 11.9. The coupling strength between radial dipole and LSP is about 2.5 times than that of orbital dipole, which is consistent with the report of C.C. Yang et al. [38]. The EQE of QWs in Ag NP region has two similar peaks and decreases monotonically with the increase of polarization angle from 0° to 90°. However, the EQE of QW at 210 nm is about twice that of QW at 270 nm. The dissipation rate at 270 nm is larger because of the edge effect.

**Figure 12:**
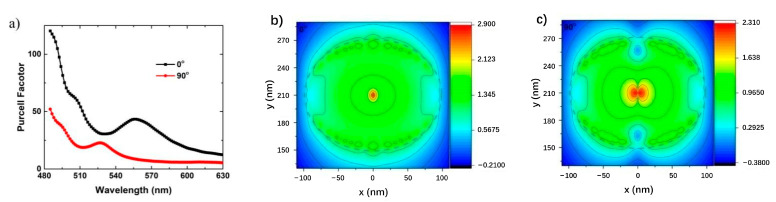
(**a**) Purcell factor curves of q-dipole with polarization angles of 0 ° and 90 ° at 210 nm. 2D mapping (log scale) of electric field distribution of q-dipole with polarization angles of (**b**) 0 ° and (**c**) 90 ° at 545 nm emission wavelength. The colored bars represent the log scale of the electric field intensity [53]. Reprinted with permission from ref. [53]. 2018, the Royal Society of Chemistry.

In order to understand the coupling process of dipoles in different polarization directions in more detail, a plane monitor is placed under the bottom of Ag NP. The curves of the Purcell factor of these dipoles with wavelength are calculated. Without losing generality, the Purcell factors of dipoles at 210 nm (under the center of the Ag NP) with polarization angles of 0° and 90° are plotted for analysis. As shown in Figure 12a, for LSP coupled with 0° and 90° dipoles, the resonance peak wavelengths are 555 nm and 525 nm, respectively. In the whole spectral range, the coupling strength between 0° angle q-dipole and LSP is greater than 90° angle q-dipole. Even so, for a 90° q-dipole, the Purcell factor can still reach about 10 at 545 nm. As shown in Figure 12b,c, a monitor as described above is used to calculate a two-dimensional (2D) mapping of the electric field distribution. It is worth noting that the LSP mode excited by a q-dipole with an angle of 0° shows the dipole mode characteristics dominated by a longer resonant peak at 555 nm, as shown in Figure 12b. Similarly, quadrupole mode characteristic dominated by a shorter resonant peak at 525 nm excited by a q-dipole with a 90° angle is observed, as shown in Figure 12c. For the q-dipole with any angle between 0° and 90°, the coupled mode can be regarded as the combination of the two basic coupled modes. It is worth noting that the electric field in Figure 12b can be enhanced by 16 times compared with the electric field in Figure 12c, which again shows that the QWs with smaller polarization angle has stronger LSP-QW coupling. Since the dipole mode coupling is stronger and it is easier to radiate light into the air [9,38], the higher EQE with smaller polarization angle discussed above can be proved. Recently, LSP mode is also explained by far-field mode [48]. The results show that the LSP mode can be well explained by combining the near-field and far-field modes. The results show that the smaller the polarization angle, the greater the Purcell factor of the dipole, and the greater the contribution to the light emission enhancement through the coupling with the low-order LSP mode.

### 3.3. Dipoles at Different Positions

In the case of orthogonal dipoles, we use the linear approximation method to distinguish the energy transferring of a single dipole and its corresponding LSP radiation and dissipation energy. Because the amplitude of z-dipole is much larger than that of x-dipole, the linear variation of dissipation energy and scattering energy of z-dipole with the total energy of z-dipole is approximately obtained. However, in many cases, the oscillation amplitude of the dipole is similar. Linear approximation may lead to large errors, for example, quantum dot (QD)-metallic NP–QW system [34,36]. In order to study the energy transferring of this kind of three body system, a perturbation method with small power value δP and its first-order asymptotic expansion is proposed. Theoretically, perturbation theory is a set of methods to study various problems in mathematics, mechanics and physics [49]. In addition, perturbation theory has been successfully applied to celestial mechanics (study of Moon–Earth–Sun system) [50] and quantum mechanics [45].

**Figure 13:**
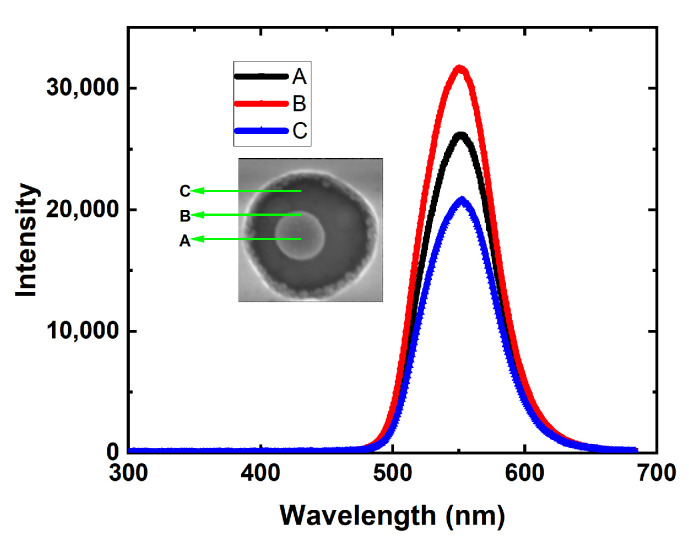
CL spectra of Ag-PhC/QW samples measured at points A, B and C. The insets show the measurement points in SEM image. Reprinted with permission from ref. [52]. 2020, Multidisciplinary Digital Publishing Institute.

The green LED wafers are in the same run as the samples in Section 2. The Ag NPs embedded in the hexagonal PhCs array holes were fabricated in the green LED by nanoimprint and lift-off techniques (labeled as Ag–PhC/QW), as described in Ref. [22] in detail. The SEM and CL measurements were carried out on the samples similar to Section 3.1. In SEM images, the diameters, height of the Ag NPs are shown as 200 and 167 nm. Figure 13 shows the CL spectra of Ag–PhC samples with QWs measured at three different points in the PhC hole. The inset shows SEM image, which marked the impinging points as A, B and C. Only one peak wavelength in each curve is about 551 nm, corresponding to the emission of QWs and/or LSPs. Electron beam (ebLSP) and quantum well (qwLSP) excite LSP. Compared with point C, the CL peak intensity of point A and point B is 1.26 times and 1.52 times that of point C, respectively. At point A of Figure 13, it is difficult for the e-beam to penetrate Ag NP with a diameter of 200 nm [53]. Because the direct emission of LSP is very weak, the strong CL emission mainly comes from the LSP excited by QW radiator under Ag NP [59]. When ebLSP is coupled with QWs at points A and B, the CL intensity is higher than that directly excited by electron beam at point C. At point C, both e-beam and QWs are outside the evanescent field of LSP, and their characteristic length is about 50 nm [17,31].

**Figure 14:**
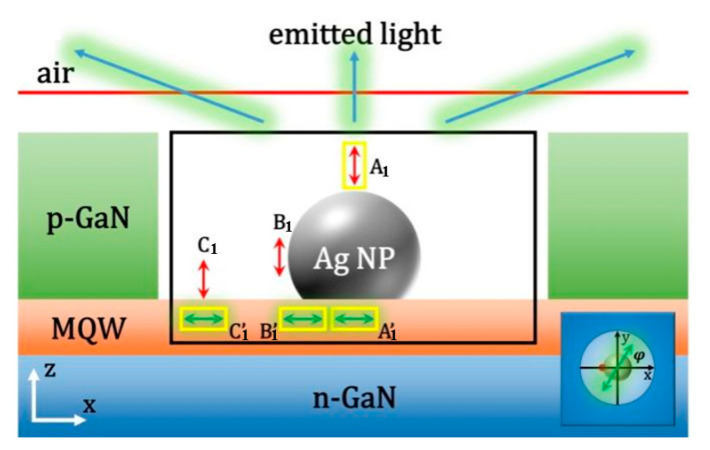
The schematic structure of 3D FDTD simulation. The black box monitor is used to collect the scattered power of the whole three-body system. The yellow transmission boxes are used to record the radiation power of z-dipole and q-dipole. The red plane monitor is used to record the power transmitted from the top surface. Red and green double-headed arrows represent e-beam and QW, respectively. The inset shows the polarization angle (φ) of the q-dipole. Reprinted with permission from ref. [52] 2020, Multidisciplinary Digital Publishing Institute.

Figure 14 shows the schematic structure in 3D-FDTD simulation. Strictly speaking, the QW is a two-dimensional structure, which is within the x–y plane. Therefore, in the simulation, it is necessary to move the q-dipole on the x–y plane, and then obtain the simulation results at different q-dipole positions through certain statistical methods. If there is no z-dipole in the simulation, due to the symmetry of the whole simulation system, the q-dipole can be placed below the Ag NP (Point A′_1_ in Figure 14) to simplify the model. When the z-dipole is added to the simulation, the symmetry of the system will be destroyed unless the z-dipole is placed at position A_1_. In order to simplify the model, the q-dipole is placed at the point where the two dipoles interact most strongly. This position is determined by the electric field distribution at 551 nm in the x–z plane at each z-dipole position. Therefore, in the following simulation of the three-body system, when the z-dipole is placed at points A_1_, B_1_ and C_1_ respectively, the q-dipole is placed at points A′_1_, B′_1_ and C′_1_.

Assuming that the e-beam itself does not radiate light, the energy radiated by the z-dipole needs to be subtracted from the three body (z-dipole-Ag-q-dipole) system. Since the power of z-dipole and q-dipole is scattered or dissipated by Ag NP, the quantum efficiency (QE) of the system can be defined as,

(13) $$\eta_{QE}=\frac{\sum_{s}\alpha_{s}\left(\lambda \right)\times P_{s}\left(\lambda \right)}{\sum_{s}P_{s}\left(\lambda \right)}$$

where the subscript ‘*s*’ represents the dipole index in the system, *α_s_*(*λ*) Is the scattering rate of the *s*th dipole, *P_s_*(*λ*) is the radiation power of the *s*th dipole. In particular, for q-dipoles, *P_s_*(*λ*) is equivalent to *Fp* due to renormalization. According to the records of the monitors in Figure 14, the sum of the scattered power $\underset{s}{\sum }\alpha_{s}\left(\lambda \right)\times P_{s}\left(\lambda \right)$, is recorded by black monitor box (*P_b_*), and *P_s_*(*λ*) is available from yellow monitor box (*P_y_*). Obviously:

(14) $$P_{b}=\sum_{s}\alpha_{s}\left(\lambda \right)\times P_{s}\left(\lambda \right)$$

Given that *α_s_* is the characterization of dipole scattering ability, it can be written as a function of *P_s_*(*λ*), namely *α_s_*(*P_s_*(*λ*)). Based on Taylor series, *α_s_*(*P_s_*(*λ*)) can be expanded to:

(15) $$\alpha_{s}\left(P_{s}\left(\lambda \right)\right)\approx \alpha_{s}^{\left(0\right)}+\alpha_{s}^{\left(1\right)}\delta P_{s}\left(\lambda \right)$$

Among them, *α_s_*^(0)^ and *α_s_*^(1)^ represent the zeroth and the first derivative of *α_s_*. In order to solve *α_s_*^(0)^ and *α_s_*^(1)^, a “small” term is added to the three body system by using the perturbation method. Because *P_s_*(*λ*) is mainly regulated by its amplitude (*A*), *P_s_*(*λ*) can be written exactly as a function *P_s_*(*λ*,*A*). In the simulation, a small change ∆*A* adds a to the q-dipole, that is, *A* becomes *A* + ∆*A*. After the second run of the simulation, *δP_s_*(*λ*,*A*) can be obtained from *P_s_*(*λ*,*A* + ∆*A*) − *P_s_*(*λ*,*A*) and Equation (14) can be rewritten as:

(16) $$P_{bBox}^{\prime }=\sum_{s}\alpha_{s}^{\prime }\left(\lambda \right)\times P_{s}^{\prime }\left(\lambda \right)\approx \sum_{s}\left(\alpha_{s}^{\left(0\right)}+\alpha_{s}^{\left(1\right)}\delta P_{s}\left(\lambda ,\;A\right)\right)\times P_{s}^{\prime }\left(\lambda \right))$$

where prime(′) indicates that the system has been “perturbed“. Then combined with linear Equations (14) and (16), *α_s_*^(0)^ and *α_s_*^(1)^ can be solved. In order to ensure that this perturbation method gives correct convergence results, ∆*A* should be small enough. Simulation results show that the calculation has good convergence under certain conditions, and ∆*A*/*A* is less than 10^−4^. The IQE and LEE of q-dipole can be obtained from Equations (1) and (2).

**Figure 15:**
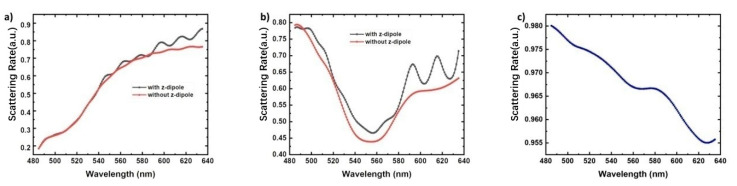
Average scattering rate spectra of the q-dipoles for Points (**a**) A, (**b**) B and (**c**) C. The calculation uses the perturbation method and weighted averages by the Fp values with from 0° to 180°. Reprinted with permission from ref. [52] 2020, Multidisciplinary Digital Publishing Institute.

The scattering and dissipation energies of the three body system are obtained by perturbation calculation. Figure 15 shows the average scattering rate spectra of q-dipoles at point A_1_, B_1_ and C_1_, respectively. All scattering rates are weighted by their *Fp* values. For point A_1_, the average scattering rate at 551 nm is 60.72%, which is greater than 59.81% without z-dipole. For longer wavelengths, *Fp* with z-dipole is much larger than *Fp* without z-dipole, as shown in Figure 15a. At point A_1_, even if the two dipoles are orthogonal to each other and the change of *Fp* is small for the case with and without z-dipoles, the dissipation can be suppressed when affected by ebLSP, which is consistent with our previous results [12]. For point B_1_, the average scattering rate at 551 nm is 47.36%, indicating that Ag NP dissipates more than half of the total radiation power. The calculated results are also 44.01% larger than those without z-dipole, indicating that the dissipation is also restrained after adding z-dipole. In addition, we found that both curves in Figure 15b have a minimum value close to 550 nm, which means that LSP symmetry breaking leads to low Fp and high energy dissipation. A resonance peak with high scattering rate appears in the short wavelength region. For point C_1_, the scattering rate is greater than 95% and changes little in the range of 485 to 635nm. Ag NP is too far away from q-dipole to dissipate energy. In addition, because the coupling of LSP to QW and e-beam is too weak, *Fp* is about 1.1, and the IQE cannot be significantly improved.

When α = 1.67, the EQE of point A_1_ and point B_1_ are 1.18 and 1.54 times that of point C_1_, respectively, which is consistent with the experimental results of 1.26 and 1.52 times. Comparing points A_1_ and B_1_ with point C_1_, the enhancement of EQE at point B_1_ is mainly due to the increase of LEE from 2.945 to 6.506%, and the increase of IQE at point A_1_ from 27.49 to 31.76%. This means that both the resonator and the antenna have a significant effect on the emission enhancement of the LSP coupled to the radiator. Reducing the thickness of metal shielding layer and effectively scattering near-field energy to far-field are the key problems. Although ebLSP is not commonly used in practical LEDs, it is an effective tool to analyze the interaction between metal nanostructures and orthogonal dipoles. The simulation results based on perturbation show that the variable separation in the many-body system will clarify the energy transfer process of SP coupling to the radiator, which is conducive to the design of efficient LED structure in the future.

## 4. Summary

In this work, many radiators coupled with LSP mechanisms were studied on different LED structures by PL, CL measurements and 3D-FDTD simulation. The conventional InGaN/GaN MQWs were simplified as five parallel dipoles. The size and morphology were optimized by the ratio of scatter and dissipation power of LSP. Considering the initial IQE of bare LED and FDTD simulations results, IQE and LEE of the LSP-coupled LED were deduced, respectively. Considering the interaction of the five dipoles and their feedback effect to LSP, LSP-dipole coupling strength enhanced and energy dissipation in Ag NPs reduced. A new LSP-QW coupling model including the enhanced dipoles was proposed to realize light emission enhancement in high EQE LEDs. The high-energy electron-induced SPs coupling with QWs were investigated. FDTD simulation analyzed the coupling mechanisms of the orthogonal dipoles and arbitrary polarized dipoles with SP in green LEDs. The linear approximation and perturbation method demonstrated the three-body system of e-beam-LSP-QW. The orthogonal dipoles coupling to LSP can suppress the metal dissipation. The z-polarized dipoles in QWs parallel to the electron beam incident direction show more coupling strength to the dipole and quadrupole modes of LSP. These results indicate that more than two polarization orientation radiators coupling to LSP or the radial polarization radiator to Ag NPs would scatter the light out and suppress the dissipation effectively.

## Data Availability

Not applicable.
